# Evolutionary diversification of the *STAYGREEN* gene family in *Nicotiana*

**DOI:** 10.3389/fpls.2026.1839826

**Published:** 2026-06-19

**Authors:** Yi Zhang, Honghai Li, Jintao Zhang, Huiyuan Ye, Weihao Wang, Maomao Hu, Xixin Zhou, Xiangli Xie

**Affiliations:** 1 College of Bioscience and Biotechnology, Hunan Agricultural University, Changsha, China; 2 China Tobacco Hunan Industrial Co., Ltd., Hunan, China; 3 School of Chemistry and Materials Science, Hunan Agricultural University, Changsha, China

**Keywords:** chlorophyll degradation, leaf senescence, *Nicotiana*, polyploidy, *STAYGREEN* proteins

## Abstract

Chlorophyll degradation underpins leaf senescence and shapes leaf maturation and quality in *Nicotiana* (tobacco). This process is orchestrated by STAYGREEN (SGR) proteins that recruit chlorophyll catabolic enzymes within chloroplasts, yet how the *SGR* gene family has diversified and been organized in the context of polyploid *Nicotiana* remains largely unresolved. Here, we performed a comprehensive genome-wide and comparative analysis of *SGR* genes across representative monocot and dicot species, with a focus on *Nicotiana*. Phylogenetic analyses revealed that *Nicotiana SGRs* belong to a conserved angiosperm *SGR* lineage but exhibit copy-number expansion consistent with a polyploid genome history, with publicly available transcriptomic data further indicating expression divergence across different tissues. Codon usage analyses indicated moderate bias and predominantly mutation-driven evolution in *Nicotiana SGRs*, in contrast to strong GC-driven selection in grasses. Conserved motif and domain analyses demonstrated a highly constrained catalytic core and structural scaffold across species, whereas terminal regions showed lineage-specific diversification. Most *Nicotiana SGR* proteins retained chloroplast targeting signals, although targeting strength and cleavage-site features varied among homologs. Structural modeling further highlights the remarkable conservation of the *SGR* fold. Together, our findings reveal that polyploid genome evolution has expanded the *Nicotiana SGR* repertoire whereas preserving its core biochemical architecture, providing an evolutionary framework for understanding how chlorophyll degradation is maintained and diversified in complex plant genomes.

## 1 Introduction

*Nicotiana* (Tobacco) is an important economic crop and a widely used model system for studying polyploidy and senescence, with considerable genetic diversity and global cultivation (Molina-Hidalgo et al., 2021). As the harvested organ in most production systems, *Nicotiana* leaves undergo a maturation process in which leaf senescence plays a central role. Senescence reshapes leaf physiology and metabolism, and chlorophyll degradation–driven yellowing is among the most visible and agronomically relevant phenotypes (Woo et al., 2019; Guo et al., 2021). Chlorophyll breakdown reflects chloroplast and photosystem remodeling and is closely linked to nutrient remobilization, which may influence *Nicotiana* maturation and quality-related traits (Domínguez and Cejudo, 2021; Wang and Grimm, 2021). Therefore, variation in the timing and pace of chlorophyll catabolism may influence maturation dynamics and quality-related traits in leaf-harvested *Nicotiana*. Understanding the genetic components underlying chlorophyll degradation is therefore essential for both senescence biology and trait improvement in *Nicotiana*.

The *SGR* gene family encodes pivotal Mg-dechelatase-recruiting proteins that orchestrate chlorophyll breakdown (Shimoda et al., 2016). SGR proteins facilitate the disassembly of light-harvesting chlorophyll a/b-binding protein complexes and recruit chlorophyll catabolic enzymes such as pheophorbide an oxygenase (PAO) and red chlorophyll catabolite reductase (RCCR) (Aubry et al., 2008; Balazadeh, 2014). Identified initially through stay-green mutants in *Festuca pratensis* that retained leaf greenness during senescence, SGR homologs have since been functionally characterized across diverse angiosperms (Vicentini et al., 1995; Thomas and Ougham, 2014). Notably, loss-of-function of SGR genes causes a stay-green phenotype by impairing chlorophyll degradation during senescence or ripening, as shown in *Arabidopsis* (*NYE1*/*SGR1*), rice (*Oryza sativa*, *OsSGR*), and tomato (*Solanum lycopersicum*, *SlSGR1*) (Jiang et al., 2007; Sakuraba et al., 2014; Uluisik et al., 2022). Plant *SGR* gene families exhibit substantial inter-species variation in copy number and functional specialization, primarily arising from lineage-specific gene duplication, whole-genome duplication, and polyploidization processes, particularly prevalent in Solanaceae (Zhou et al., 2011; Barchi et al., 2019; Luo et al., 2023). Because *SGR* function is tightly coupled with chloroplast processes, features such as subcellular targeting signals (e.g., chloroplast transit peptides) and the stability of conserved protein regions may determine whether its function is maintained across species or among duplicated copies (Thomas and Howarth, 2000; Sakuraba et al., 2015). Whereas the physiological roles of *SGR*s are well-documented, how these genes have structurally and functionally diverged in complex polyploid genomes remains a key question in evolutionary genomics.

*Nicotiana* is an allotetraploid species (2*n* = 4*x* = 48) formed by ancestral hybridization between *N. sylvestris* and *N. tomentosiformis* (Sierro and Ivanov, 2020). The *Nicotiana* polyploid origin produced two subgenomes and many homologous gene pairs (Lim et al., 2004). Therefore, numerous *Nicotiana* gene families contain multiple copies, some of which were retained, lost, or diversified after polyploidization (Bombarely et al., 2012). Polyploid genomes may also experience additional duplications and structural changes; gene copy number and sequence variation can be further reshaped over time (Soltis and Soltis, 1999). These features make *Nicotiana* a valuable system for studying how senescence-related gene families evolve in a complex genome. Chlorophyll degradation occurs in chloroplasts and is coordinated with chloroplast dismantling during senescence (Tamary et al., 2019; Domínguez and Cejudo, 2021). In polyploid *Nicotiana*, duplicated *SGR* copies may have been differentially retained and diverged in sequence features relevant to chloroplast localization and protein function, potentially contributing to variation in yellowing dynamics that matter for leaf-harvested quality. Although *SGR* functions have been well studied in several model plants and crops, the *SGR* gene family in *Nicotiana* has not been systematically characterized at the genome level. A more integrated analysis that combines phylogeny, molecular evolution, and conserved structural features is still needed.

In this study, we conducted a genome-wide comparative analysis of the *Nicotiana SGR* gene family to clarify its evolutionary diversification and potential functional divergence. Using curated *SGR* candidates from available *Nicotiana* genome resources, along with representative monocot and dicot homologs, we reconstructed the phylogenetic relationships of *SGR* genes across angiosperms. Our analyses revealed that *Nicotiana SGR*s belong to a conserved angiosperm lineage but have undergone copy-number expansion consistent with a polyploid genome history. Publicly available transcriptomic data further suggest expression divergence among duplicated *SGR* homologs across different tissues. Synonymous codon usage analyses indicated moderate codon usage bias and predominantly mutation-driven evolutionary patterns in *Nicotiana SGR*s. Conserved motif, domain, chloroplast-targeting, and structural analyses showed that the core *SGR* protein architecture is highly conserved, whereas terminal regions and targeting features exhibit copy- or lineage-specific variation. Together, these findings provide an evolutionary framework for understanding how polyploidy has shaped the *Nicotiana SGR* gene family whereas maintaining conserved chlorophyll-degradation functions.

## 2 Materials and methods

### 2.1 Data preparation

Protein sequences annotated as members of the *SGR* gene family were retrieved from the National Center for Biotechnology Information (NCBI) database using keyword searches including “Stay-Green”, “SGR”, “NYE1”, “SGR-like” and associated locus tags. This candidate-retrieval strategy was based on previous genome-wide *SGR* family studies and on the established nomenclature of functionally characterized *SGR* homologs in model plants and crops (Sakuraba et al., 2015; Uluisik et al., 2022; Luo et al., 2023). All available plant *SGR* homologs with complete or near-complete open reading frames were downloaded in FASTA format. To ensure accurate classification, each sequence was cross-checked against its NCBI Gene, Protein and Taxonomy records.

Candidate sequences were further filtered to remove redundant protein entries, alternative isoforms representing the same gene, very short partial sequences and low-quality annotations. Proteins shorter than 150 amino acids were excluded as likely incomplete fragments, because canonical plant SGR proteins generally contain a conserved Stay-Green domain and are typically much longer than this threshold. Candidate proteins were further validated by domain annotation, and proteins containing the conserved Stay-Green domain were retained as bona fide *SGR*/*SGRL* candidates for downstream analyses.

Taxonomic identifiers were used to assign each protein to its corresponding genus, species and higher-level lineage according to the NCBI taxonomy database. To improve the robustness of cross-taxon comparative analyses, only genera with at least five non-redundant *SGR*/*SGRL* protein sequences were retained. This genus-level inclusion threshold was defined in this study to ensure sufficient representation for phylogenetic, codon-usage, motif and domain comparisons. Based on this criterion, eight representative plant genera were included: *Arabidopsis*, *Glycine*, *Nicotiana*, *Oryza*, *Populus*, *Setaria*, *Solanum* and *Triticum*. These taxa represent major angiosperm lineages, including dicots and monocots, and include widely studied model plants and crop species.

For reproducibility, detailed metadata were recorded for all *SGR*/*SGRL* sequences used in this study, including protein accession, gene symbol or locus tag, GeneID, species name, genus assignment, protein length, database source and use in downstream analyses. Cultivar, ecotype or accession information was recorded when it was available in the original genome annotation or NCBI source record. When such information was not provided in the source record, it was recorded as “not specified in source record” rather than inferred from external sources. The complete sequence metadata are provided in **Supplementary Table 1**.

Genome resources used for synteny analysis, candidate mapping and expression profiling were also documented. For each genome resource, the species name, cultivar/ecotype or accession information, assembly version, annotation version, genome FASTA file, GFF/GTF annotation file, protein FASTA file, CDS or cDNA file, database source and analysis purpose were recorded. These genome and annotation resources are summarized in **Supplementary Table 2**.

### 2.2 Phylogenetic analyses

Phylogenetic analyses of *SGR* genes analyzed in this study were performed using a suite of bioinformatics tools. Multiple sequence alignment was carried out with MAFFT (option: --auto) (Katoh and Standley, 2013), followed by alignment trimming using trimAl (v1.4.rev15; parameters: -phylip -automated1) (Capella-Gutiérrez et al., 2009). All resulting phylogenetic trees were visualized and annotated in iTOL (Letunic and Bork, 2016) and graphically refined in Adobe Illustrator 2025 (Adobe Inc.).

### 2.3 Synteny and gene duplication analysis

To investigate the genomic basis of SGR family expansion and to infer orthologous and paralogous relationships, synteny analyses were performed using chromosome-level genome assemblies and corresponding gene annotation files. For *Nicotiana* tabacum, the Nitab-v4.5 genome assembly and Edwards2017 gene annotation were used as the reference dataset. Intragenomic synteny analysis was first conducted for *N. tabacum* to identify duplicated *NtSGR* and *NtSGRL* gene pairs retained within collinear genomic blocks. Interspecific synteny comparisons were further performed between *N. tabacum* and representative related species, including *N. sylvestris*, *N. tomentosiformis* and *Solanum lycopersicum*, to evaluate conserved orthologous relationships among SGR homologs.

Genome-wide collinearity was inferred using the One Step MCScanX module implemented in TBtools-II (Chen et al., 2023), with default parameters except that the E-value threshold for sequence similarity searches was set to 1e-10 and the maximum number of retained hits was set to 5. For each species, genome FASTA and GFF/GTF annotation files from the same assembly version were used to ensure consistency between chromosome identifiers and gene coordinates. Collinear blocks and gene-pair relationships were extracted from the resulting MCScanX collinearity files. *NtSGR* and *NtSGRL* gene identifiers were then mapped to the collinearity output to identify SGR-containing collinear blocks and syntenic SGR gene pairs. Gene duplication modes, including whole-genome/segmental, tandem, proximal and dispersed duplication, were inferred from MCScanX/TBtools duplicate-gene classification outputs where available.

### 2.4 Public RNA-seq data processing and expression profiling

To examine whether duplicated *NtSGR* and *NtSGRL* genes show evidence of regulatory divergence, publicly available RNA-seq data were used to profile tissue-level expression patterns. RNA-seq run accessions, BioProject accessions and tissue annotations were obtained from the sample information table of a published tobacco expression atlas. Representative RNA-seq runs were selected to cover major tobacco tissues, with up to three biological runs retained for each tissue when available. In total, 39 RNA-seq runs from 1 BioProject were selected, covering leaf, root, stem, flower, petal, sepal, blade, lamina, midrib, shoot, axillary shoot, trichome and seedling leaf samples. The complete list of RNA-seq run accessions, BioProject accessions and tissue annotations is provided in **Supplementary Table 3**.

Raw sequencing data were retrieved from the Sequence Read Archive using SRA Toolkit. SRA files were converted to FASTQ format using fasterq-dump with the --split-files option. Reads were quality-filtered using fastp with the following parameters: -q 20, -u 30 and -l 50. Both paired-end and single-end datasets were processed according to the layout of each run. Only quality-filtered reads were used for downstream expression profiling.

Because the objective was to compare relative expression patterns among curated *NtSGR/NtSGRL* candidates rather than to perform transcriptome-wide quantification, a curated *NtSGR/NtSGRL* cDNA reference was constructed from the Nitab-v4.5 cDNA dataset. Candidate NtSGR/NtSGRL proteins were first mapped to Nitab-v4.5 protein models by BLASTP (Mahram and Herbordt, 2015), and the corresponding Nitab-v4.5 cDNA sequences were extracted as the target reference. Quality-filtered RNA-seq reads were mapped to this curated *NtSGR/NtSGRL* cDNA reference using CoverM (Aroney et al., 2025) in contig mode with minimap2-sr as the mapper. Relative transcript abundance was estimated using the TPM method, together with read count and RPKM outputs for inspection. Mapping was performed with a minimum read identity of 90% and a minimum aligned-read fraction of 80%.

For each RNA-seq run, TPM values were extracted for all *NtSGR/NtSGRL* cDNA reference sequences. Tissue-level expression values were calculated as the mean TPM across biological runs assigned to the same tissue. The resulting expression matrix was transformed as log2(TPM + 1) and visualized as a heatmap in R using pheatmap. Row-scaled heatmaps were used to compare relative expression patterns among *NtSGR/NtSGRL* genes across tissues. The expression analysis was interpreted as tissue-level relative expression profiling of curated *NtSGR/NtSGRL* candidates.

### 2.5 Codon usage analysis of SGR Genes

Codon usage bias parameters of SGR coding sequences were calculated using CodonW (version 1.4.4). All CDSs were extracted in FASTA format and validated to ensure correct reading frames. For each gene, the effective number of codons (ENC), GC content at the third codon position (GC3s), codon adaptation index (CAI), and codon bias index (CBI) were computed.

### 2.6 Motif identification using MEME

Conserved motifs in *SGR* protein sequences were identified using the MEME Suite (version 5.5) (Bailey et al., 2015). A total of 128 full-length SGR protein sequences from eight representative plant species were used as input. The resulting motifs, including consensus sequences, E-values, and motif positions, were extracted from the MEME output and visualized using TBtools (Chen et al., 2023) and custom R scripts. Motif architecture diagrams were generated by mapping each motif to its corresponding protein sequence and aligning motif positions across species to examine conservation, motif order, and lineage-specific diversification.

### 2.7 Domain annotation using InterProScan

Protein sequences of SGR candidates were subjected to domain and family annotation using InterProScan (version 5.75-106.0). The analyses were run in standalone mode with the Pfam, SUPERFAMILY and CDD databases enabled, and the output was generated in TSV format. InterPro entries (IPR IDs) and Pfam accessions were used to identify conserved domains. Proteins containing the Stay-Green domain (InterPro entry IPR024438; Pfam family PF12638) were retained as bona fide SGR proteins.

### 2.8 Subcellular localization prediction

Subcellular localization of SGR protein sequences was predicted using TargetP-2.0 (version 2.0; plant mode) (Emanuelsson et al., 2007). The analysis generated probabilities for five possible targeting categories: chloroplast transit peptides (cTP), signal peptides (SP), mitochondrial targeting peptides (mTP), thylakoid lumen peptides (lTP), and OTHER (no targeting peptide). Proteins were assigned to the localization category with the highest prediction score. For sequences predicted to contain a cTP, TargetP also provided the putative cleavage site (CS) position and confidence scores. Predictions were extracted and summarized across species to identify lineage-specific differences in plastid targeting.

### 2.9 Protein AlphaFold3 modeling

The three-dimensional structures of the *Nicotiana* SGR protein sequences analyzed in this study were constructed and refined using the AlphaFold Server, which employs the latest AlphaFold 3 model (Abramson et al., 2024). Multiple structure alignments across different taxa were performed using mTM-Align (Dong et al., 2018).

RMSD values were calculated using the align function in PyMOL 2.5.2 to measure global structure similarity, which performs atom-by-atom superposition based on backbone Cα atoms and minimizes the distance through least-squares fitting. The reported RMSD corresponds to the average displacement across all aligned residues. Alignments were carried out under default parameters. Structural visualization and figures were also generated in PyMOL 2.5.2.

### 2.10 Statistical analysis

All statistical analyses and data visualizations were performed using R software version 4.4.1 (www.r-project.org), along with a suite of specialized packages. Core R packages included dplyr for data manipulation, and ggplot2 for general-purpose plotting. All figures were formatted for high-resolution output in PDF formats and finalized using *Adobe Illustrator* 2025 (Adobe Inc.) to ensure publication quality.

## 3 Results

### 3.1 Phylogenetic distribution and lineage expansion of the *SGR* gene family

To examine the evolutionary distribution of the *SGR* gene family across plant lineages, we retrieved SGR protein sequences from the NCBI database. We analyzed genera with more than five available sequences. Eight representative genera, *Arabidopsis*, *Glycine*, *Nicotiana*, *Oryza*, *Populus*, *Setaria*, *Solanum*, and *Triticum* were included in downstream analyses. Among them, *Solanum* contained the most significant number of *SGR* genes (30), followed by *Nicotiana* (20). *Glycine* and *Populus* each encoded 17 *SGR* genes, slightly exceeding *Arabidopsis* (16). *Triticum* and *Setaria* possessed 14 and 9 genes, respectively, whereas *Oryza* contained the fewest (7).

**Figure 1:**
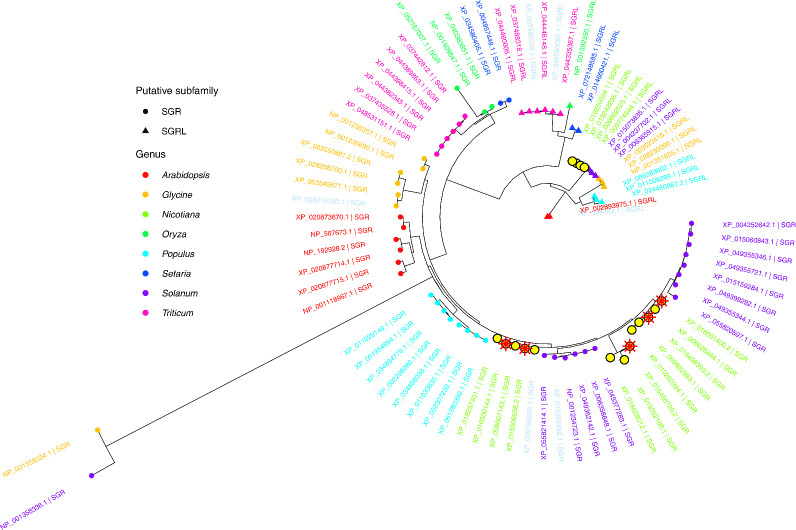
Phylogenetic classification of SGR-family amino acid sequences. Maximum-likelihood phylogeny was constructed from the curated SGR-family protein set using IQ-TREE 2. The tree is shown in a circular layout with tip labels oriented for readability. Tip-label colors indicate taxonomic groups, and tip shapes indicate putative SGR or SGRL phylogenetic clades inferred from reference sequences. *Nicotiana* members are highlighted with yellow circles, and *N. tabacum* members are marked with red stars.

A circular phylogenetic tree constructed from all SGR protein sequences revealed strong lineage-specific clustering consistent with the established evolutionary relationships among the eight genera, with high bootstrap support at most nodes (**Figure 1**). Distinct color labels denote the source genus of each SGR sequence. Using known *SGR* and *SGRL* proteins as reference anchors, the phylogenetic tree resolved two major *SGR*-family lineages corresponding to putative *SGR* and *SGRL* clades. *Nicotiana* members were distributed across these clades, indicating that representatives of both SGR-family lineages are present in the *Nicotiana* dataset. Because phylogenetic placement alone does not provide direct functional validation, these assignments were treated as putative subfamily classifications and were used primarily as an evolutionary framework for subsequent analyses.

### 3.2 Syntenic relationships and duplication patterns of *NtSGR*/*NtSGRL* genes

To investigate whether the expansion of the tobacco SGR family was associated with gene duplication and conserved genomic context, we performed intragenomic and interspecific synteny analyses using MCScanX/TBtools. The analysis focused on nine curated *NtSGR*/*NtSGRL* candidates identified from the Nitab-v4.5 reference.

Duplicate-gene classification showed that the *NtSGR*/*NtSGRL* candidates were associated with multiple duplication modes. Two genes, Nitab4.5_0000847g0060.1 and Nitab4.5_0004701g0060.1, were classified as WGD/segmental duplicates, whereas Nitab4.5_0001714g0050.1 and Nitab4.5_0001714g0060.1 were classified as tandem duplicates. Nitab4.5_0000140g0110.1 was classified as a dispersed duplicate, whereas four candidates were not assigned to a duplication category in the TBtools duplicate-gene classification output (**Supplementary Tables 4**, **S5**; **Supplementary Figure 1**).

Intragenomic collinearity analysis in *N. tabacum* identified one *NtSGR*/*NtSGRL* syntenic pair between Nitab4.5_0000847g0060.1 and Nitab4.5_0004701g0060.1. This pair was located within a collinear block between Nt12 and Nt16, with a pairwise similarity E-value of 2e-160. The duplicated status and collinear relationship of these two genes suggest that WGD/segmental duplication contributed to the retention of at least part of the *NtSGR*/*NtSGRL* repertoire in the tobacco genome.

To further infer conserved orthologous relationships, we compared SGR-containing genomic regions between *N. tabacum* and related species. In total, 15 interspecific syntenic pairs involving *NtSGR*/*NtSGRL* genes were detected, including six pairs between *N. tabacum* and *N. sylvestris*, three pairs between *N. tabacum* and *N. tomentosiformis*, and six pairs between *N. tabacum* and *Solanum lycopersicum*. Four tobacco candidates—Nitab4.5_0000847g0060.1, Nitab4.5_0004701g0060.1, Nitab4.5_0000140g0110.1 and Nitab4.5_0001714g0050.1—were located in conserved interspecific syntenic blocks (**Supplementary Table 6**; **Supplementary Figure 1**). Notably, Nitab4.5_0000847g0060.1 and Nitab4.5_0004701g0060.1 were both syntenic with Nsyl04g014040-1.1, Ntom04g013710-1.1 and Solyc04g063240.3.1, supporting conserved orthologous relationships across Solanaceae and parental-lineage genomes.

### 3.3 Tissue-level expression divergence of *NtSGR/NtSGRL* genes

To further evaluate whether retained NtSGR/NtSGRL copies show evidence of regulatory divergence, we profiled their tissue-level expression using publicly available RNA-seq datasets. A total of 39 RNA-seq runs covering 13 tobacco tissues were analysed, with three runs retained for each tissue, including axillary shoot, blade, flower, lamina, leaf, seedling leaf, midrib, petal, root, sepal, shoot, stem and trichome. Reads were mapped to the curated *NtSGR*/*NtSGRL* cDNA reference, and relative transcript abundance was summarized as TPM values.

The resulting expression heatmap revealed clear tissue-biased expression patterns among the nine curated *NtSGR*/*NtSGRL* candidates (**Supplementary Figure 2**). Several genes showed broad expression across vegetative tissues. For example, Nitab4.5_0002539g0020.1 displayed relatively high expression in photosynthetic and leaf-associated tissues, including midrib, shoot, seedling leaf, lamina and leaf, suggesting a potential role in leaf maturation or chlorophyll turnover. Nitab4.5_0000847g0060.1 and Nitab4.5_0004701g0060.1 also showed relatively stronger expression in blade, axillary shoot and seedling leaf, consistent with tissue-preferential deployment of *SGR*-related functions in vegetative organs.

In contrast, other *NtSGR*/*NtSGRL* candidates exhibited more restricted or organ-biased expression. Nitab4.5_0001714g0050.1 and Nitab4.5_0001714g0060.1 showed pronounced enrichment in trichome, with Nitab4.5_0001714g0060.1 also showing elevated expression in floral tissues such as petal, flower and sepal. Nitab4.5_0010544g0020.1 displayed relatively high expression in petal, root and trichome, whereas Nitab4.5_0011435g0010.1 was more strongly expressed in root and flower. By contrast, Nitab4.5_0008133g0020.1 showed low relative abundance across most tissues, with only weak tissue-specific signals.

Together, these expression profiles indicate that *NtSGR*/*NtSGRL* copies are not uniformly expressed across tobacco tissues. Instead, retained copies exhibit distinct tissue-level expression patterns, supporting regulatory divergence among duplicated *SGR*-family members. Although these data do not establish functional roles directly, the observed expression divergence provides candidate evidence for potential subfunctionalization or tissue-specific deployment of chlorophyll-degradation regulators in polyploid tobacco.

### 3.4 Comparative analysis of codon usage bias among *SGR* genes across plant lineages

**Figure 2:**
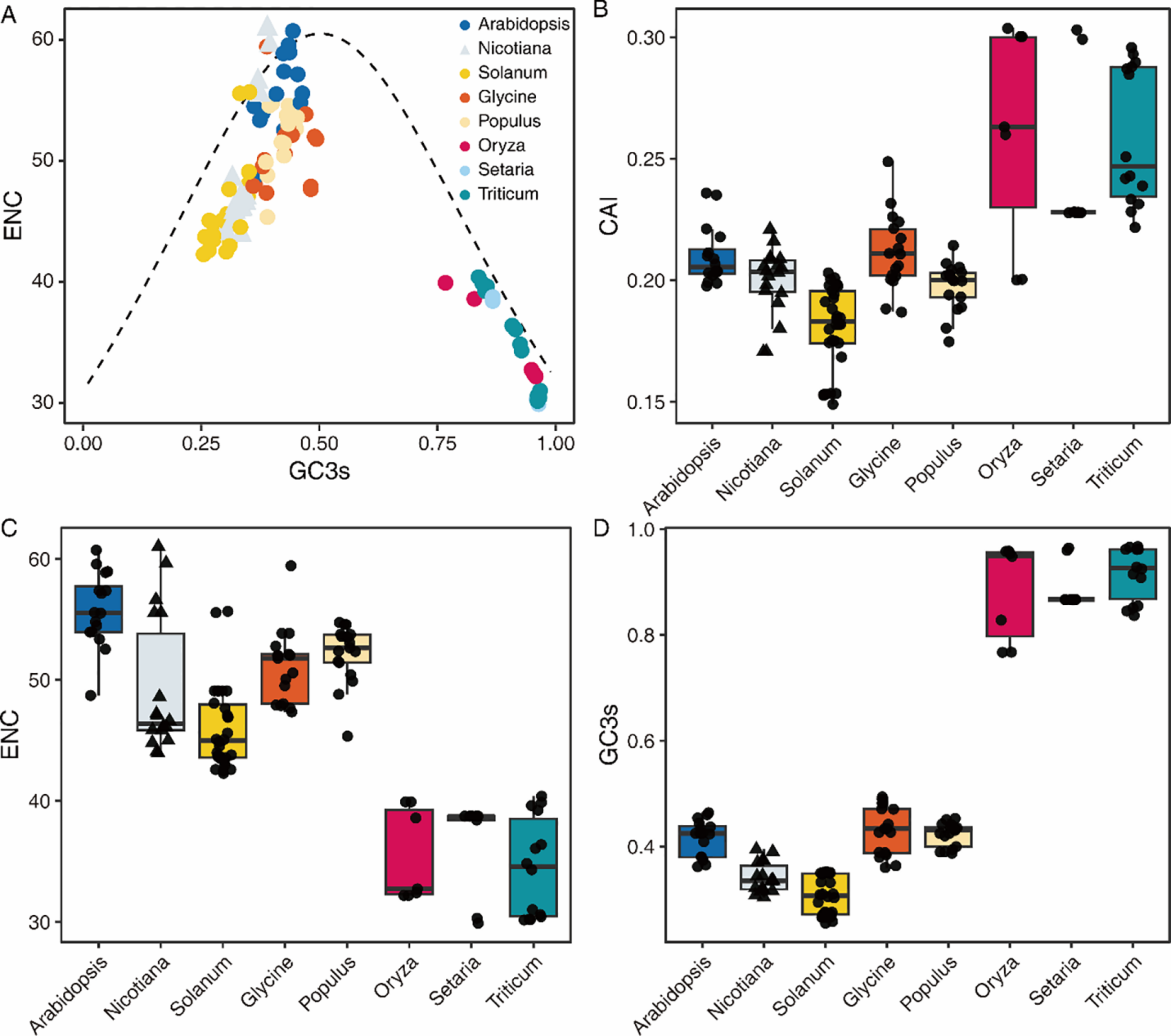
Codon usage patterns of SGR genes across representative plant groups. **(A)** Nc-plot showing the relationship between the effective number of codons (ENC) and GC content at the third codon position (GC3s) for SGR coding sequences. The dashed line represents the expected ENC under neutral mutation pressure. Points indicate individual genes and are colored by plant group, with Nicotiana highlighted by triangles. **(B)** Distribution of codon adaptation index (CAI) values across plant groups. Boxes indicate the interquartile range, horizontal lines indicate medians, and whiskers represent 1.5× the interquartile range. Individual genes are shown as points. **(C)** Effective number of codons (ENC) across plant groups. Individual genes are shown as points, with *Nicotiana* highlighted. **(D)** GC3s variation across plant groups. Boxes indicate the interquartile range, horizontal lines indicate medians, and whiskers represent 1.5× the interquartile range.

To investigate synonymous codon usage patterns in the *SGR* gene family, we calculated codon usage indices for 128 SGR coding sequences from eight representative plant species using CodonW, including the effective number of codons (ENC), GC content at the third codon position (GC3s), and codon adaptation index (CAI) (**Figure 2**; **Supplementary Figure 3**; **Supplementary Table 7**). Across all species, ENC values ranged from approximately 30 to 61, indicating substantial variability in codon usage bias within the family. GC3s values spanned more than threefold (0.26–0.97), and CAI values ranged from 0.15 to 0.30, consistent with generally weak to moderate translational adaptation.

Apparent lineage-specific differences were observed. Among dicots, *Nicotiana* exhibited relatively weak codon usage bias, with an average ENC of 49.2 ± 5.65, which was lower than that of *Arabidopsis* (55.8 ± 3.07) and *Populus* (52.1 ± 2.44), but higher than that of *Solanum* (46.0 ± 3.48). *Nicotiana* SGR genes also displayed moderately low GC3s (0.341 ± 0.028) and overall GC content (0.398 ± 0.011), indicating a preference for A/U-ending codons consistent with an AT-rich genomic background. The mean CAI of *Nicotiana* (0.200 ± 0.014) was comparable to that of *Populus* but slightly lower than that of *Arabidopsis* and *Glycine*, suggesting modest optimization for efficient translation (**Figure 2**).

In contrast, monocots such as *Oryza*, *Setaria*, and *Triticum* showed strikingly different codon usage patterns, characterized by extremely high GC3s and low ENC values (**Figures 2C, D**). Their *SGR* genes exhibited extremely high GC3s values (0.883–0.918) and markedly low ENC values (34–37), together with elevated CAI scores. These characteristics reflect strong preferences for GC-ending codons and a more pronounced codon usage bias, likely driven by lineage-specific GC enrichment and stronger translational selection in grass genomes.

Nc-plot analysis further supported these observations. Most *Nicotiana SGR* genes clustered near the expected ENC–GC3s curve, indicating that codon usage is dominated by mutational pressure and nucleotide composition. In monocots, however, many *SGR* genes fell well below the theoretical curve, suggesting that selection contributes significantly to shaping codon choice. Collectively, these results show that *Nicotiana SGR* genes exhibit moderate codon usage bias, AT-rich codon preferences, and weak translational selection. In contrast, monocot *SGR* genes display strong GC-driven optimization, reflecting distinct evolutionary pressures across plant lineages.

### 3.5 Conserved motif architecture of the *SGR* gene family

**Figure 3:**
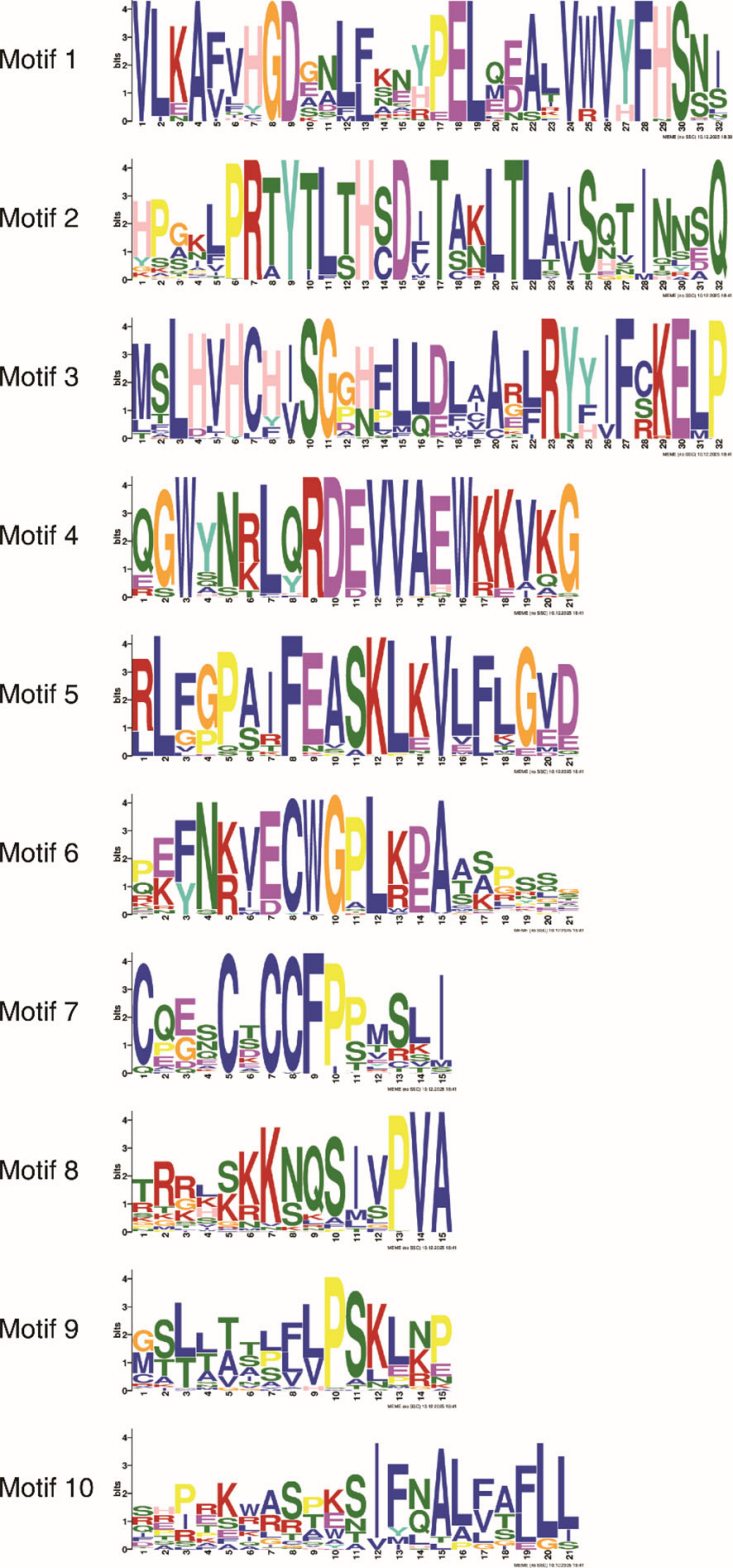
Conserved motif composition of SGR proteins across representative plant groups. Ten motifs identified by MEME are shown in different colors and labeled as Motif 1 to Motif 10. Motif positions are displayed according to their locations within each protein sequence. Protein length is indicated by the scale bar.

MEME analysis identified ten statistically significant motifs across the 128 *SGR* protein sequences from eight plant species (**Figure 3**). These motifs, ranging from 14 to 32 amino acids in length, were consistently detected in both monocots and dicots, indicating that *SGR* proteins share a highly conserved domain architecture (**Supplementary Figure 4**).

Motifs 1 and 2 were the most conserved, with the lowest E-values and present in more than 85% of all sequences. These two motifs form the catalytic core of the *SGR* domain and were enriched in hydrophobic, charged, and catalytic residues (H, D, E, W) known to mediate interactions within the chlorophyll degradation complex. Their nearly invariant spacing and central placement across all species highlight strong structural constraints on this region.

Motifs 3–6 constituted a secondary conserved block flanking the catalytic core. Their broad conservation across monocots and dicots suggests roles in stabilizing the *SGR* protein fold and supporting interactions with components of the chlorophyll catabolic machinery, including PAO, RCCR, and LHCII. The preserved motif order and spacing further suggest strong purifying selection to maintain functional integrity.

In contrast, motifs 7–10 showed greater lineage-specific variability and were predominantly located in the N- and C-terminal regions. These shorter, more flexible motifs often contained cysteine-rich or charged residues, suggesting possible roles in regulatory modulation, subcellular targeting, or species-specific protein–protein interactions. Monocot *SGR* proteins, particularly those from *Triticum* and *Setaria*, exhibited more frequent motif substitutions or expansions, indicating diversification at accessory regions.

*Nicotiana* SGR proteins displayed a typical dicot-like motif profile: all central motifs (1–6) were conserved entirely with minimal sequence variation, and no motif loss or rearrangement was observed. Variation occurred only in accessory motifs (7–10), suggesting subtle lineage-specific fine-tuning that does not alter the overall SGR architecture.

Together, these results support a dual-domain architectural model for plant SGR proteins. A highly conserved catalytic core (Motifs 1–2) underpins the enzyme’s essential function in chlorophyll degradation, whereas a conserved structural scaffold (Motifs 3–6) stabilizes the protein and mediates key molecular interactions. By contrast, terminal accessory motifs (Motifs 7–10) are more variable and likely contribute to regulatory diversification across lineages. This conserved-core, variable-periphery organization underscores strong evolutionary constraints on SGR function whereas allowing adaptive divergence in peripheral regions.

### 3.6 Comparative domain architecture and gene family expansion of *SGR* proteins

**Figure 4:**
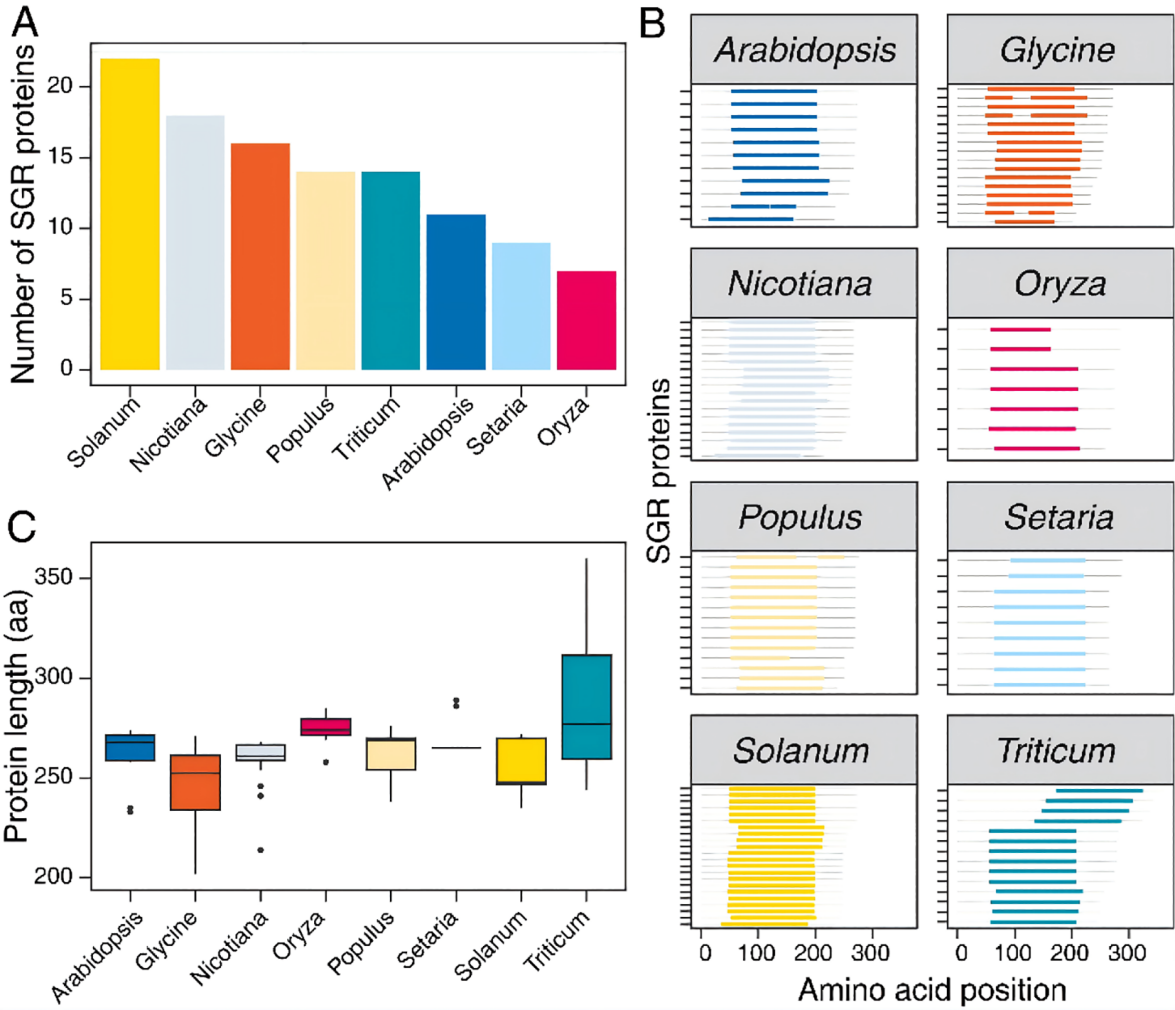
Comparative features of SGR proteins across representative plant groups. **(A)** Number of proteins containing the conserved Stay-Green domain identified in each plant group based on InterProScan annotation. **(B)** Domain architecture of SGR proteins. Gray bars indicate full protein length, and colored regions indicate the Pfam-annotated Stay-Green domain PF12638. **(C)** Distribution of SGR protein lengths across plant groups. Boxes indicate the interquartile range, horizontal lines indicate medians, and whiskers represent 1.5× the interquartile range.

To investigate lineage-specific differences in the *SGR* gene family, we quantified proteins containing the conserved *SGR* domain (PF12638/IPR024438) across eight representative plant species. The number of *SGR* proteins varied markedly among taxa (**Figure 4A**). Dicotyledonous species such as *Solanum*, *Nicotiana*, *Glycine*, and *Populus* generally encoded 6–10 *SGR* proteins, whereas *Arabidopsis* contained slightly fewer. In contrast, monocot species, including *Oryza*, *Setaria*, and *Triticum* harbored noticeably expanded *SGR* repertoires, consistent with lineage-specific gene duplication events. These observations indicate that whereas the presence of *SGR* genes is conserved across angiosperms, the extent of family expansion differs substantially between monocots and dicots.

To compare the structural characteristics of SGR proteins, we examined the domain organization of all InterProScan-annotated sequences (**Figure 4B**). Despite differences in total protein length, nearly all *SGR* proteins contained a single centrally located *SGR* domain (PF12638), with highly consistent domain position and length across species. This structural uniformity reflects strong evolutionary constraints on the catalytic and regulatory functions of *SGR* proteins in chlorophyll degradation.

Variation among proteins was primarily confined to their terminal regions. Several monocot *SGR* proteins exhibited modestly extended C-terminal areas, whereas many dicot proteins displayed more compact architectures. Importantly, all species retained the characteristic *SGR* motif block, underscoring its essential and evolutionarily conserved role in chlorophyll catabolism.

We further compared the distributions of total protein lengths across species (**Figure 4C**). Most *SGR* proteins ranged from 230 to 300 amino acids (aa), with dicots clustering tightly around 250–270 aa. Monocots displayed a broader length distribution, including a distinct subset exceeding 280 aa, consistent with the extended terminal regions observed in **Figure 2**. Despite these differences, the relatively narrow overall length range supports the view that *SGR* proteins exhibit strong structural conservation, with monocot-specific extensions likely reflecting lineage-dependent functional elaboration.

### 3.7 Variation in chloroplast targeting of *SGR* proteins

To determine the subcellular localization patterns of *SGR* proteins, we performed TargetP analysis across representative monocot and dicot species. The majority of SGR proteins were predicted to contain chloroplast transit peptides (cTP), consistent with their essential role in chlorophyll degradation. In most dicots, such as *Arabidopsis*, *Solanum*, *Nicotiana*, *Populus*, and *Glycine*—70–95% of *SGR* sequences were assigned to the cTP class. In contrast, monocots, including *Oryza*, *Setaria*, and *Triticum*, showed similarly high proportions of predicted chloroplast localization (**Supplementary Figure 5**). *Nicotiana* displayed a dominant cTP signature overall, though a small subset of sequences was classified as OTHER, indicating potential diversification lineage-specific diversification in subcellular targeting.

Comparative analysis of cTP prediction scores revealed distinct lineage-level patterns (**Supplementary Figure 6**). In monocot species, *Oryza* and *Setaria* exhibited significantly higher median cTP scores, indicating stronger chloroplast-targeting signals. Dicots showed greater variation: *Arabidopsis* and *Glycine* consistently exhibited strong cTP signatures, whereas *Nicotiana* and *Solanum* displayed weaker, more variable targeting strengths. These differences point to subtle evolutionary divergence in N-terminal targeting sequence architecture, most noticeably within *Nicotiana*.

**Figure 5:**
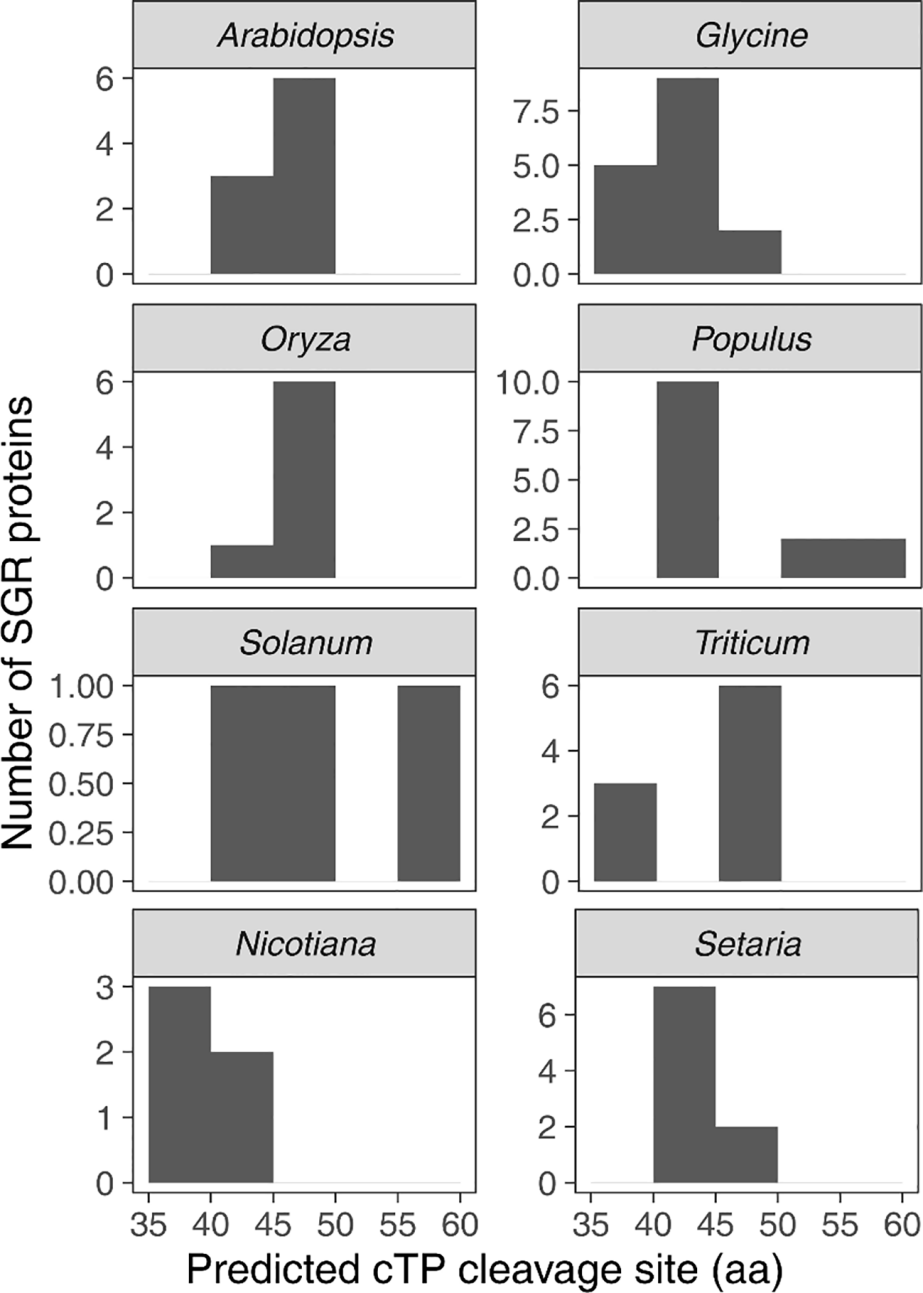
Distribution of predicted chloroplast transit peptide cleavage-site positions in SGR proteins. Histograms show the predicted chloroplast transit peptide cleavage-site positions generated by TargetP-2.0.

Analysis of predicted chloroplast transit peptide cleavage sites further indicated strong structural conservation. Across all species, *SGR* transit peptides were typically 45–80 amino acids in length, consistent with canonical chloroplast import requirements (**Figure 5**). Monocots exhibited highly constrained cleavage-site distributions, whereas dicots showed broader variation, with *Nicotiana* and *Solanum* displaying the greatest flexibility. This suggests that, although the core features required for chloroplast targeting remain conserved, dicot lineages may be experiencing relaxed constraints or early-stage diversification in transit peptide architecture.

Collectively, these results reveal that whereas chloroplast localization is broadly conserved across angiosperms, *Nicotiana SGR* proteins exhibit more variable and moderately attenuated chloroplast-targeting features. This may reflect lineage-specific functional innovation or shifts in regulatory or degradative contexts that differentiate *Nicotiana* from other dicots and monocots.

### 3.8 Structural comparison between *Nicotiana SGR* and the bacterial 7Y5Y template

**Figure 6:**
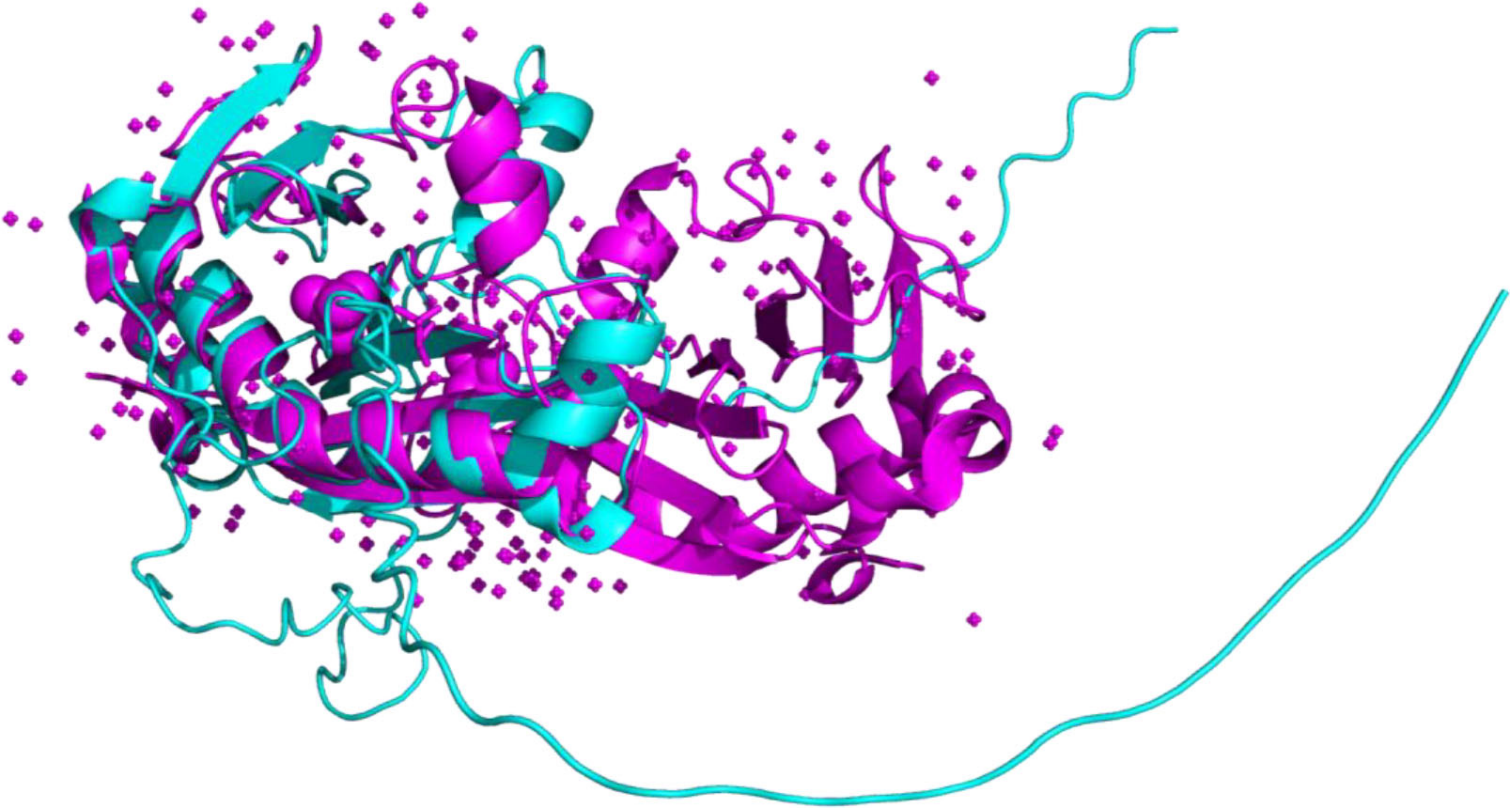
Structural alignment between a *Nicotiana* SGR AlphaFold3 model and the bacterial SGR crystal structure. The Nicotiana SGR model was generated using AlphaFold3 and aligned with the bacterial SGR crystal structure PDB 7Y5Y using PyMOL. *Nicotiana* SGR is shown in blue, and 7Y5Y is shown in purple. The RMSD value and number of aligned Cα atoms are indicated in the figure. The alignment yielded an RMSD of 0.72 Å across 111 aligned Cα atoms.

To assess the structural conservation of *SGR* proteins across evolutionary lineages, we compared the AlphaFold3-predicted *Nicotiana SGR* structure with the experimentally resolved crystal structure of an SGR homolog from *Anaerolineae* bacterium (PDB ID: 7Y5Y) (**Figure 6**). Full-length structural alignment revealed striking similarity between the two proteins, with an exceptionally low RMSD of 0.72 Å across 111 Cα atoms. After excluding flexible loop regions that showed poor alignment, the optimized superposition yielded an RMSD of 0.72 Å across 111 Cα atoms.

This exceptionally low RMSD demonstrates that the overall *SGR* fold, including the characteristic central α/β core of the Stay-Green domain is highly conserved between dicot plants and distantly related bacteria. Despite substantial primary sequence divergence, the core domain architecture remains nearly superimposable, indicating that the structural framework required for chlorophyll catabolism has been maintained over deep evolutionary time. This conservation suggests strong functional constraints on the SGR fold, reinforcing the notion that key structural features of chlorophyll-degrading enzymes were established early and preserved across diverse lineages.

## 4 Discussion

Elucidating the evolutionary organization of chlorophyll catabolic regulators is fundamental to understanding how conserved senescence pathways are shaped by genome evolution. *SGR* proteins are core components of chlorophyll degradation and senescence-associated leaf yellowing, and their biological roles have been well established in *Arabidopsis*, rice, and tomato. In this study, we showed that *Nicotiana SGR* genes formed a conserved yet expanded gene family shaped by polyploid genome history. Although *Nicotiana SGR*s retained the canonical structural features required for chlorophyll degradation, they exhibited copy-level divergence in sequence composition, predicted chloroplast-targeting properties, and evolutionary constraints. Together, these findings indicated that *Nicotiana* genome evolution have not altered the fundamental biochemical role of *SGR*, but instead influenced how this function was deployed across multiple homologous copies.

Plant lineage history plays a central role in determining gene family composition, and polyploidy is widely recognized as a major driver of gene retention in plants (Van de Peer et al., 2021; Heslop-Harrison et al., 2023). Polyploid genomes undergo differential gene retention following whole-genome duplication events, and genes involved in regulatory networks and developmental transitions are preferentially retained over multiple rounds of duplication (Edger and Pires, 2009; Tasdighian et al., 2017). Comparative genomic analyses across polyploid species have shown that senescence-associated gene families, including transcription factors and metabolic regulators, exhibit marked copy number expansion relative to their diploid relatives (Emery et al., 2018; Song et al., 2020). Transcriptomic profiling in polyploid wheat and cotton revealed that many retained senescence-related homologs display tissue-specific or developmental-stage-specific expression patterns, consistent with subfunctionalization after duplication (Adams, 2007; Hovav et al., 2008). These observations suggest that regulatory complexity in senescence pathways may scale with genome ploidy level, and that multiple gene copies can partition ancestral functions across distinct spatial or temporal contexts. By contrast, our phylogenetic analyses place *Nicotiana* S*GR*s within the conserved angiosperm *SGR* lineage whereas revealing a larger and more complex *SGR* repertoire than is typically observed in diploid species. In the broader phylogenetic context, compact and well-supported *SGR* clades in *Triticum*, *Solanum*, and *Glycine* suggest lineage-specific expansion and retention of closely related homologs, whereas the broader distribution of *Arabidopsis* and *Nicotiana SGR*s indicates greater sequence divergence within these genera. The separation of monocot *SGR*s from dicot homologs further supports lineage-specific diversification of the *SGR* family across angiosperms. Similar expansion of senescence-related regulators has been reported in other polyploid crops, suggesting that duplication-associated retention is common for genes involved in major developmental transitions (Fan et al., 2018; Wu et al., 2019; Evans et al., 2022). In *Nicotiana*, this expanded repertoire implies that chlorophyll degradation is unlikely to be governed by a single *SGR* gene, but rather by multiple homologs that may act in a coordinated manner.

The synteny analysis provides genomic-context evidence supporting the expansion and retention of *NtSGR/NtSGRL* genes in polyploid tobacco. In particular, the intragenomic collinear pair Nitab4.5_0000847g0060.1–Nitab4.5_0004701g0060.1, both classified as WGD/segmental duplicates, suggests that large-scale duplication or polyploidy-associated segmental retention contributed to the tobacco *SGR*-family repertoire. The identification of tandem and dispersed duplicate categories further indicates that *SGR*-family diversification in tobacco was not driven by a single duplication mechanism, but rather by multiple genome-evolutionary processes.

The interspecific synteny results further support conserved orthologous relationships between tobacco SGR-family members and homologs in related species. Several *NtSGR/NtSGRL* genes retained syntenic relationships with homologs in *N. sylvestris*, *N. tomentosiformis* and *S. lycopersicum*, indicating that part of the tobacco *SGR* repertoire can be traced to conserved Solanaceae genomic regions and parental-lineage contexts. However, not all curated *NtSGR*/*NtSGRL* candidates were detected in *SGR*-containing collinear blocks, suggesting that some copies may have experienced lineage-specific rearrangement, local duplication, divergence beyond synteny detection, or incomplete assignment by the current genome annotation and collinearity workflow. Thus, the tobacco *SGR* family appears to reflect both conserved orthologous retention and lineage-specific duplication after polyploidization.

Beyond genomic-context conservation, the expression profiles further suggest that retained *NtSGR*/*NtSGRL* copies may have diverged at the regulatory level after duplication. Although the core *SGR* domain and motif architecture are broadly conserved, the RNA-seq heatmap revealed distinct tissue-biased expression patterns among individual *NtSGR*/*NtSGRL* genes. Some copies were preferentially expressed in vegetative or leaf-associated tissues, whereas others showed stronger expression in trichome, root or floral tissues. Such expression partitioning is consistent with regulatory divergence after gene duplication and may reflect partial subfunctionalization of retained copies in the polyploid tobacco genome. These observations support a model in which polyploidy expanded the *NtSGR*/*NtSGRL* repertoire, whereas subsequent regulatory divergence allowed different copies to be deployed across distinct tissue contexts.

Although *Nicotiana* harbors an expanded set of *SGR* homologs, this expansion does not correspond to substantial divergence in the functional determinants of *SGR* proteins. Across plant species, even single amino-acid substitutions within conserved regions of *SGR* proteins are sufficient to generate stay-green phenotypes, underscoring the sensitivity of *SGR* activity to structural integrity (Jiang et al., 2007; Park et al., 2007; Barry et al., 2008). Consistent with these observations, all *Nicotiana SGR*s analyzed here preserve the canonical *SGR* domain, conserved motif organization, and a highly similar three-dimensional fold. These patterns support a conserved-core, variable-periphery model for plant *SGR* proteins, in which Motifs 1–2 likely maintain the essential chlorophyll-degradation function, Motifs 3–6 contribute to structural stability and molecular interactions, and more variable terminal motifs may facilitate regulatory diversification among lineages or retained copies. In contrast, sequence divergence among *Nicotiana SGR*s is primarily observed outside these conserved regions. Such divergence is unlikely to abolish chlorophyll-degrading activity, but may influence regulatory properties, protein stability, or interaction capacity. Moreover, the exceptionally low RMSD between representative plant and bacterial *SGR*-like proteins further indicates that the central α/β Stay-Green fold has been maintained over deep evolutionary time, suggesting strong structural constraints on the molecular framework required for chlorophyll catabolism. These observations suggest that duplication in the *Nicotiana* lineage has preserved *SGR* proteins with intact biochemical function, whereas allowing variation in regions more tolerant to sequence change.

Chlorophyll degradation occurs in chloroplasts, and *SGR* proteins are generally characterized as plastid-localized factors across plant species (Hörtensteiner, 2009; Sakuraba et al., 2012). In agreement with this established biology, most *Nicotiana SGR*s are predicted to contain chloroplast transit peptides. However, predicted targeting strength and cleavage-site features vary among *Nicotiana SGR* copies. Transit peptides are known to evolve rapidly and are defined largely by compositional properties rather than strict sequence conservation (Bruce, 2000; Lee et al., 2008). Therefore, the variability in predicted cleavage sites among *Nicotiana SGR*s aligns with this notion of rapid evolution and compositional definition (Teng et al., 2012). Instead, it may translate into quantitative differences in protein import efficiency, processing, or accumulation within chloroplasts. Similar variation in plastid targeting has been proposed to influence the timing and extent of chloroplast-associated processes in other plant systems (Teng et al., 2012). In *Nicotiana*, such differences may contribute to variation in senescence progression and leaf yellowing dynamics. At the level of coding-sequence evolution, synonymous codon usage patterns of *Nicotiana SGR*s are consistent with the broader genomic context of Solanaceae rather than strong gene-specific translational optimization (Sablok et al., 2013; Anwar et al., 2021). This contrasts with grasses, where GC3 enrichment and pronounced codon bias are commonly observed (Tatarinova et al., 2010). Given that *SGR* expression is typically induced during senescence rather than maintained constitutively, limited evidence for strong translational selection is not unexpected (Quax et al., 2015; Frumkin et al., 2018). Together with the high conservation of functional domains, these results indicate that sequence-level evolution of *Nicotiana SGR*s primarily reflects genome-wide mutational and compositional biases. Functional differentiation among *SGR* copies is therefore more likely driven by regulatory divergence and protein-level properties than by extensive optimization of coding sequences (Gu et al., 2005).

In summary, our study demonstrates that the *Nicotiana SGR* gene family represents a conserved but expanded component of the plant senescence machinery shaped by polyploid genome evolution. By integrating phylogenetic analysis, protein feature characterization, and comparative evolutionary context, we show that *Nicotiana SGR*s retain intact biochemical features required for chlorophyll degradation whereas diverging in regions associated with regulation and chloroplast targeting. These findings suggest that senescence and leaf yellowing in *Nicotiana* are regulated by the combined action of multiple *SGR* homologs rather than by a single dominant gene. Our results provide an evolutionary perspective on how gene duplication and constraint jointly influence key senescence regulators and highlight the importance of gene family context when dissecting the genetic basis of leaf aging and quality-related traits. Future gene-specific functional analyses will be needed to determine whether individual *NtSGR*/*NtSGRL* copies have acquired distinct physiological roles during tobacco leaf maturation and senescence.

## Data Availability

The original contributions presented in the study are included in the article/**Supplementary Material**. Further inquiries can be directed to the corresponding author.
