# Supplementary material for: Evolutionary diversification of the STAYGREEN gene family in Nicotiana
Source: Front Plant Sci. 2026 Jun 19;17:1839826. doi: 10.3389/fpls.2026.1839826 (PMC13330579; doi:10.3389/fpls.2026.1839826)
Supplement: Supplementary file 2 [file Table2.docx]

**Supplementary Information for**

Evolutionary diversification of the *STAYGREEN*  gene family in *Nicotiana*

**Authors: Yi Zhang^1^, Honghai Li^1^, Jintao Zhang^2^, Huiyuan Ye^2^, Weihao Wang^1^, Maomao Hu^1^, Xixin Zhou^3*^, Xiangli Xie^1*^**

**Author Affiliations:**

^1^College of Bioscience and Biotechnology, Hunan Agricultural University, Changsha 410019, China

^2^China Tobacco Hunan Industrial Co., Ltd., Hunan 410019, China

^3^School of Chemistry and Materials Science, Hunan Agricultural University, Changsha 410128, China

*Corresponding Author: Xixin Zhou; Xiangli Xie
E-mail: 152924447@qq.com; xiexl@hunau.edu.cn

**Supplementary Figure**


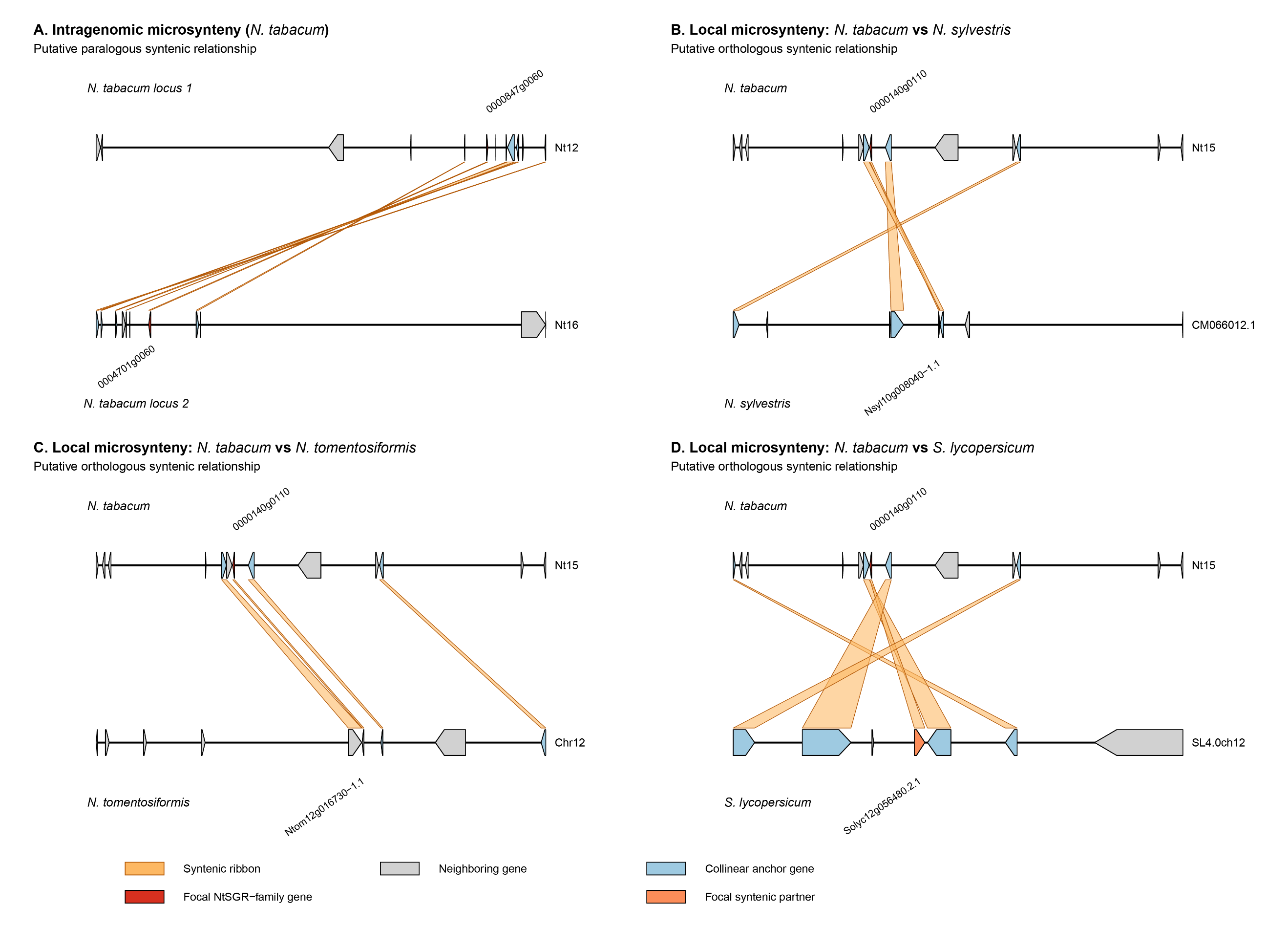


**Figure S1.** Local microsynteny of NtSGR-family loci in N. tabacum and related species.

(A) Intragenomic microsynteny between two NtSGR-family loci in N. tabacum. (B–D) Interspecific microsynteny between N. tabacum NtSGR-family loci and corresponding syntenic regions in N. sylvestris, N. tomentosiformis and Solanum lycopersicum. Gene arrows indicate gene orientation. Orange ribbons indicate syntenic links between collinear genes. Grey arrows indicate neighboring genes, blue arrows indicate collinear anchor genes, red arrows indicate focal NtSGR-family genes, and orange arrows indicate focal syntenic partner genes.


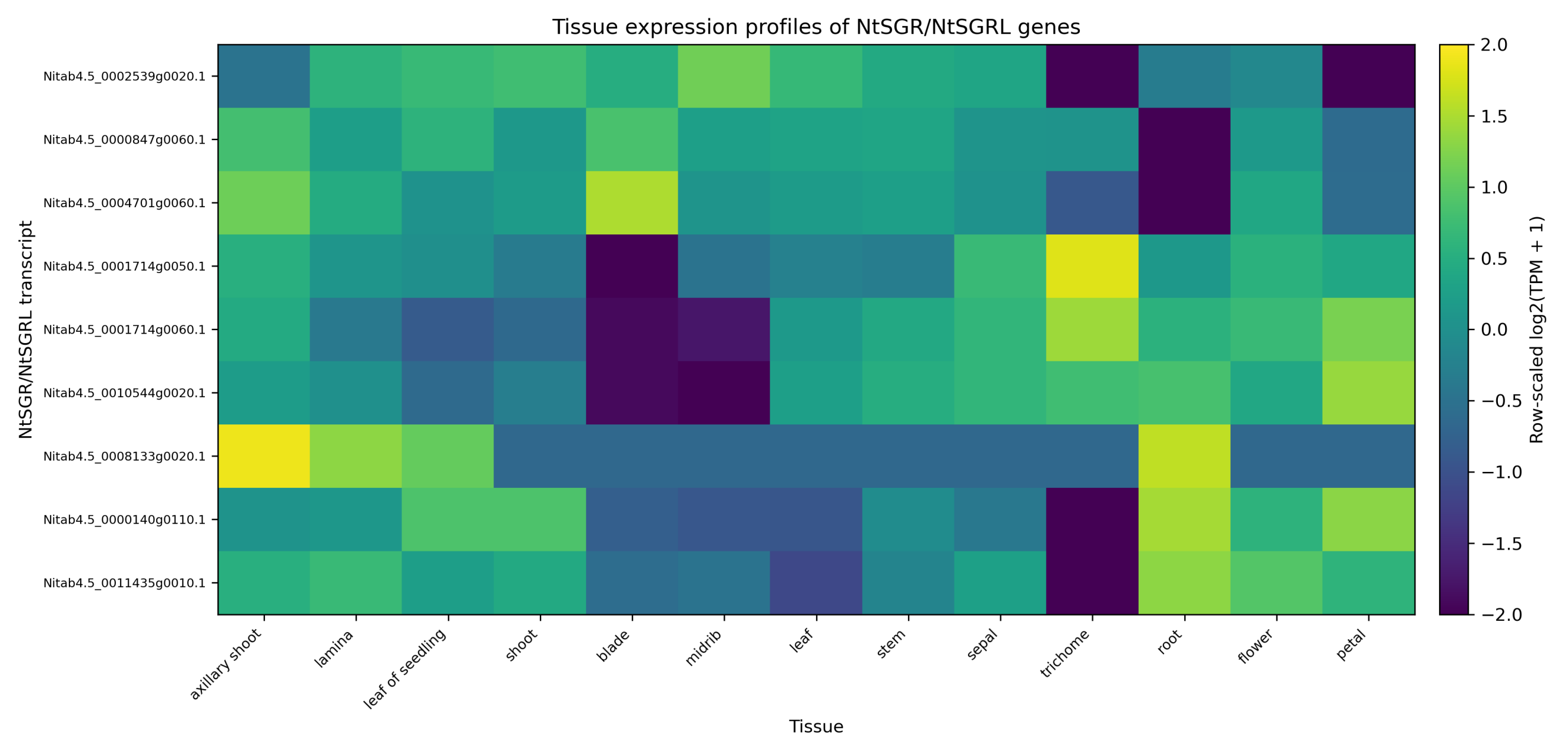


**Figure S2.** Tissue-level expression profiles of *NtSGR*/*NtSGRL* genes across tobacco tissues.

Heatmap showing relative expression patterns of nine curated *NtSGR*/*NtSGRL* cDNA candidates across 13 tobacco tissues. Public RNA-seq reads from 39 runs were mapped to the curated *NtSGR*/*NtSGRL* cDNA reference, and TPM values were calculated for each run. Tissue-level values represent the mean TPM of three runs per tissue. Expression values were transformed as log2(TPM + 1) and row-scaled to highlight relative tissue-preferential expression patterns for each gene. Rows represent *NtSGR*/*NtSGRL* transcripts and columns represent tissues.


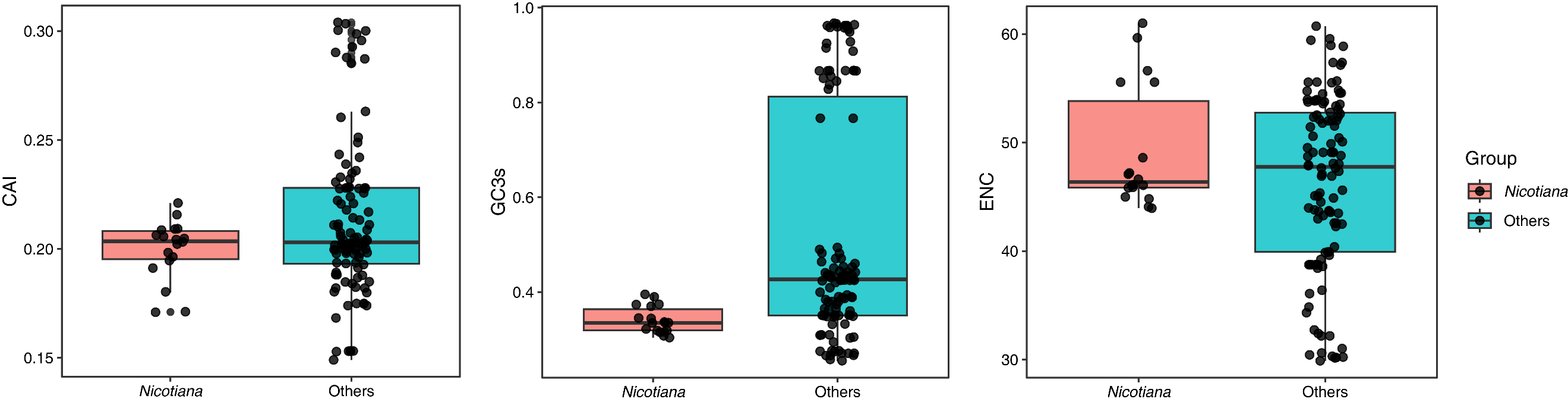


**Figure S3.** Comparison of CAI, GC3s and ENC between *Nicotiana* and all other species.


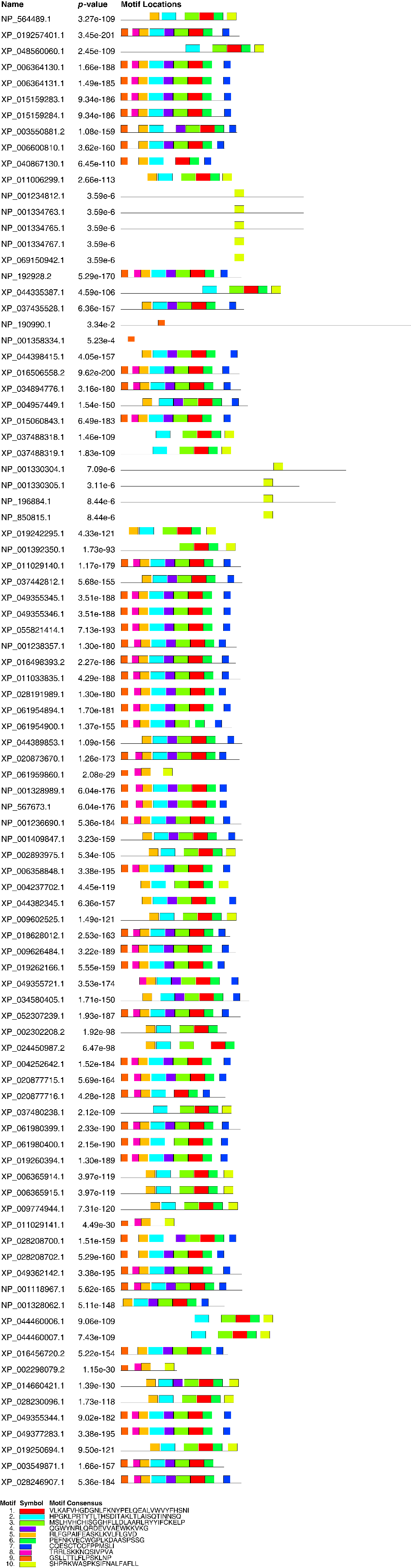


**Figure S4.** Motif Locations of SGR protein across plant species.
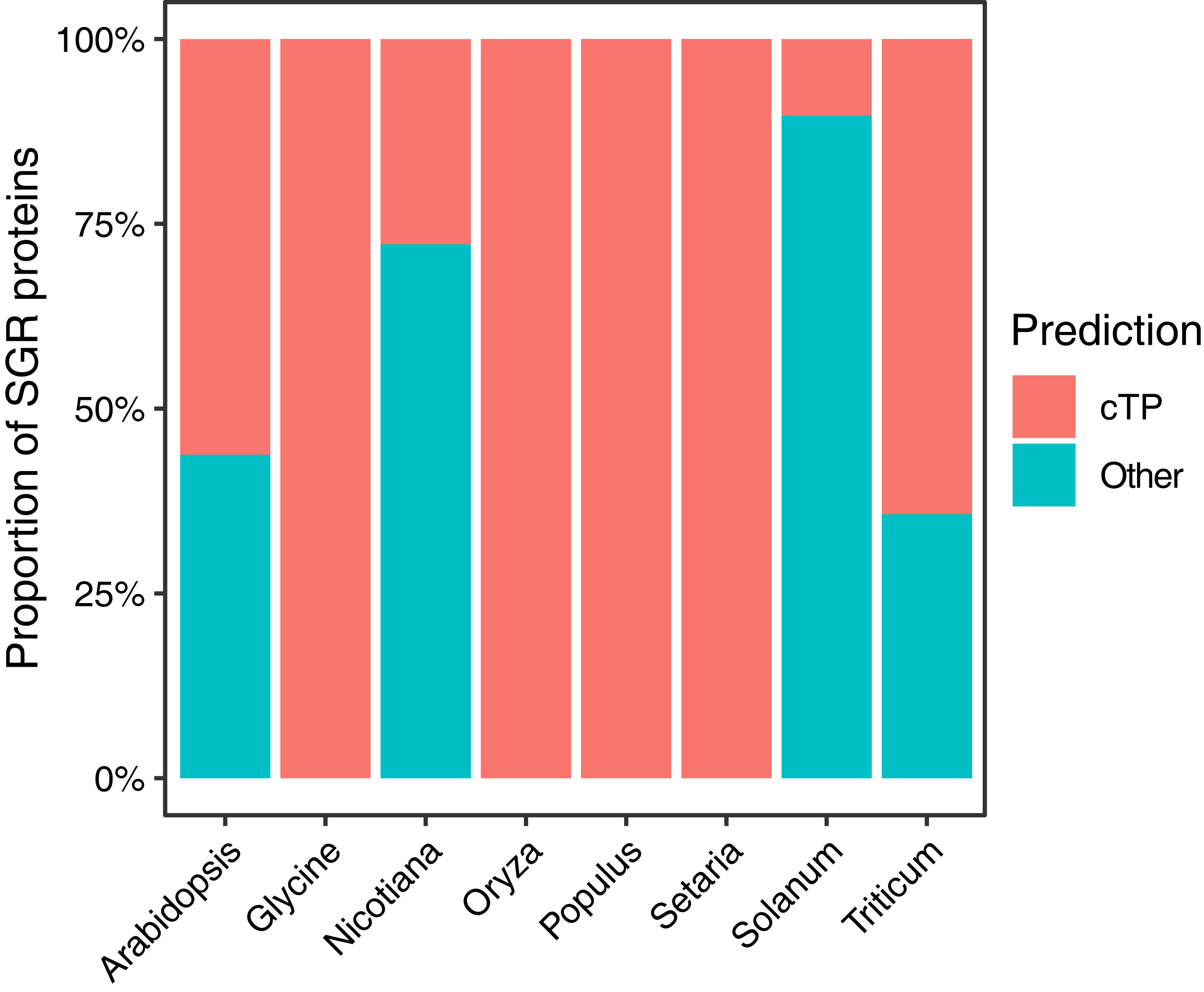


**Figure S5. Distribution of TargetP-predicted localization classes among SGR proteins across plant species.** Barplots show proportional representation of TargetP prediction categories (cTP, SP, mTP, OTHER). Most SGR proteins were predicted to contain a chloroplast transit peptide (cTP), with Nicotiana showing moderate but consistent cTP presence.


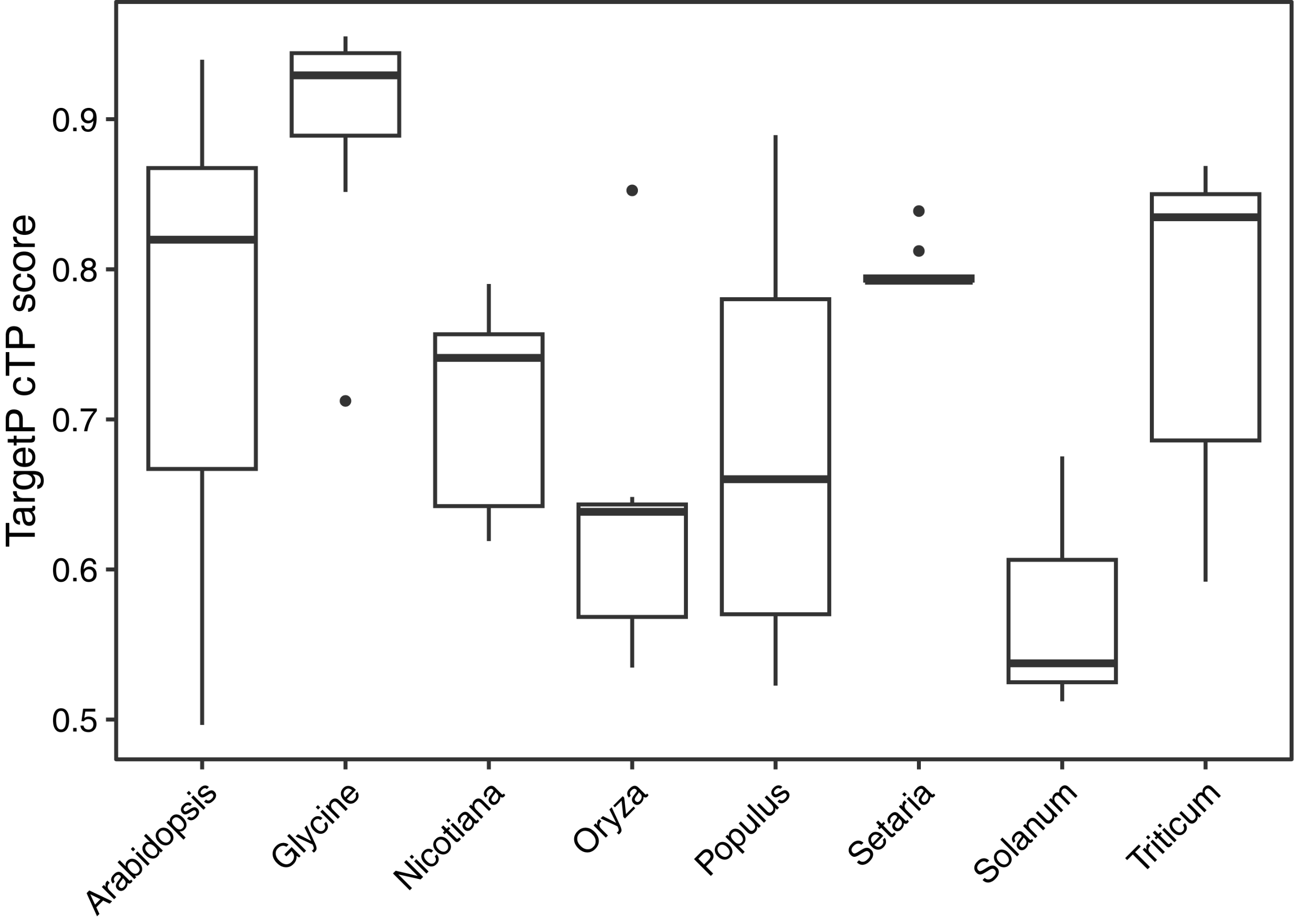


**Figure S6. Comparison of chloroplast transit peptide (cTP) prediction scores across species.** Boxplots summarize cTP scores for proteins predicted as chloroplast-targeted (cTP). Monocots generally exhibited higher cTP scores than dicots, while Nicotiana displayed intermediate values and greater score variability.

**Supplementary Tables**

**Table S7.** **Mean codon usage indices of SGR genes across eight plant groups.** Values represent mean ± standard deviation. CAI: codon adaptation index; ENC: effective number of codons; GC3s: GC content at the third codon position.

| **Group** | **n** | **CAI** | **ENC** | **GC3s** | **GC** |
| --- | --- | --- | --- | --- | --- |
| ***Arabidopsis*** | 16 | 0.210 ± 0.0119 | 55.8 ± 3.07 | 0.416 ± 0.0342 | 0.442 ± 0.0121 |
| ***Nicotiana*** | 18 | 0.200 ± 0.0139 | 49.2 ± 5.65 | 0.341 ± 0.0283 | 0.398 ± 0.0110 |
| ***Solanum*** | 30 | 0.181 ± 0.0170 | 46.0 ± 3.48 | 0.308 ± 0.0362 | 0.394 ± 0.0261 |
| ***Glycine*** | 17 | 0.211 ± 0.0158 | 51.1 ± 3.07 | 0.431 ± 0.0461 | 0.442 ± 0.0215 |
| ***Populus*** | 17 | 0.198 ± 0.00993 | 52.1 ± 2.44 | 0.423 ± 0.0221 | 0.443 ± 0.00919 |
| ***Oryza*** | 7 | 0.261 ± 0.0453 | 35.4 ± 3.82 | 0.883 ± 0.0918 | 0.699 ± 0.0238 |
| ***Setaria*** | 9 | 0.244 ± 0.0322 | 36.8 ± 3.79 | 0.888 ± 0.0419 | 0.668 ± 0.0275 |
| ***Triticum*** | 14 | 0.259 ± 0.0285 | 34.5 ± 4.09 | 0.918 ± 0.0504 | 0.693 ± 0.0147 |
